# Informing drinkers: Can current UK alcohol labels be improved?

**DOI:** 10.1016/j.drugalcdep.2018.07.032

**Published:** 2018-11-01

**Authors:** Anna K.M. Blackwell, Katie Drax, Angela S. Attwood, Marcus R. Munafò, Olivia M. Maynard

**Affiliations:** a UK Centre for Tobacco and Alcohol Studies, School of Psychological Science, University of Bristol, 12a Priory Road, Bristol, BS8 1TU, UK; b MRC Integrative Epidemiology Unit (IEU), University of Bristol, Oakfield House, Oakfield Grove, Bristol, BS8 2BN, UK; c Department of Psychology, University of Bath, 10 West, Bath, BA2 7AY, UK

**Keywords:** Alcohol labeling, Alcohol health warnings, Alcohol units, Consumer knowledge, Public health

## Abstract

**Background:**

Alcohol labeling provides a relatively low-cost, population-level approach to providing information about alcohol’s content and harms.

**Method:**

We conducted an online between-subjects experiment with two tasks to examine the impact of alcohol labels (n = 1884). In one task, participants were randomized to view one of four different unit labels (including labels currently used by the alcohol industry and novel labels which provide more information about how the number of units relates to recommended drinking guidelines). We assessed participants’ accuracy of estimating weekly serving limits of alcohol. In a second task, participants were randomized to view one of eight health warnings (which varied according to message content, specificity, and framing). We assessed the motivation to quit after viewing the health warning.

**Results:**

Accuracy of estimating weekly serving limits of alcohol was greater for participants who viewed novel unit labels compared to the industry standard labels. Motivation to drink less was higher amongst participants who had viewed both cancer and negatively framed messages, compared to mental health and positively framed messages.

**Conclusion:**

Existing unit labels used by the alcohol industry can be improved; the inclusion of unit information per serving and how these relate to low-risk drinking guidelines may be important for facilitating consumer understanding. Health warning labels should be included alongside units to provide consumers with information about the harms associated with alcohol and discourage riskier drinking behavior.

## Highlights

• Drinkers underestimated the number of drinks that constituted 14 units.
• Unit understanding was greater for novel unit labels compared to industry labels.
• Motivation to drink less was higher for cancer and negatively-framed messages.

## 1 Introduction

Alcoholic drinks differ widely in their strength and size, and consumers often underestimate their alcohol intake (Kerr and Stockwell, 2012). More than 10 million adults in England drink more than the current UK low-risk guidelines (Health and Social Care Information Centre, 2015) and poor communication of alcohol strength and alcohol-related harm means that people may not be aware of the risks.

Alcohol units (i.e., 10 ml or 8 g of pure alcohol in the UK) are a method of providing information about the strength of an alcoholic drink in relation to its serving size. A similar method used in Australia, known as ‘standard drink’ labeling, has been shown to effectively improve drinkers’ estimates of their consumption and help them monitor their intake (Kerr and Stockwell, 2012; Osiowy et al., 2015). However, there are also potential unintended consequences of alcohol strength labeling. Focus groups with Australian undergraduate students found that they often used this information to select high strength, low-cost alcohol (Jones and Gregory, 2009) and recent experimental research indicates that labeling alcoholic beverages as lower in strength increases the amount consumed (Vasiljevic et al., 2018).

This highlights the important role of designing health warnings on alcohol labels that can increase public awareness of the potential harms of excessive alcohol consumption (Knai et al., 2015). Current warnings are often limited to the dangers of drinking when pregnant but could be extended to include other health conditions, such as the risk of cancer and depression. Although the impact of health warnings on drinking behavior is less clear (Miller et al., 2016; Scholes‐Balog et al., 2012; Wilkinson et al., 2009; Wilkinson and Room, 2009), evidence from their implementation on tobacco products indicates that they may be effective (Hammond, 2011). Research with Australian drinkers found that specific rather than general health warnings were rated as more believable and effective (Miller et al., 2016; Pettigrew et al., 2014), while both positively (e.g., ‘Reduce your drinking to reduce your risk of cancer’) and negatively (e.g., ‘Warning: alcohol increases your risk of cancer’) framed messages may play a useful role in supporting message believability, personal relevance and facilitating purchasing decisions (Jarvis and Pettigrew, 2013; Pettigrew et al., 2014).

Some effort has been made to improve understanding of alcohol’s content and risks. In 2011, the alcohol industry agreed to a voluntary Responsibility Deal with the UK Government, which included labeling at least 80% of their products with unit content, low-risk guidelines, pregnancy warnings and responsibility statements (Department of Health, 2011). However, a review found alcohol labels in the UK often fell short of best practice based on the small size of the health information and its placement on the back of products (Petticrew et al., 2016). The UK Chief Medical Officers’ low-risk guidelines were updated in 2016 (Department of Health, 2016), but a month after their release, a survey of the UK drinkers found that only 8% of participants knew the new guidelines (Rosenberg et al., 2017). Over a year later, a review of over 300 alcohol products found that only one label included the updated information (Alcohol Health Alliance UK, 2017). In 2017, the Portman Group, an organization established by the alcohol industry to promote responsible drinking, removed low-risk guidelines from their list of key labeling elements (Portman Group, 2017). Given failures in voluntary agreements, statutory regulation for alcohol labels has been recommended in a report by the UK House of Lords European Union Committee (European Union Committee, 2015).

Here we examine the influence of unit labels and health warnings on drinkers’ understanding, attitudes and behavioral intentions regarding drinking and examine optimal methods of delivering this information on labels. This is important given the current political interest in alcohol labeling and can be used to guide the development of label designs that can effectively support consumers.

## 2 Methods

### 2.1 Study design

This was an online between-subjects experimental study with two tasks. In the first task, participants were pseudo-randomized to view one of the four-unit labels where the primary outcome measure was the accuracy of estimating weekly serving limits of alcohol. Two of the unit labels displayed industry standard information typically seen on alcohol products and the other two displayed novel methods of presenting unit information (see 2.3.3 for details). In the second task, participants were randomized to view one of eight health warnings (which varied in their specificity, framing and health message, see 2.3.4 for details). Motivation to drink less was the primary outcome measure. Support for alcohol labeling policies before and after the experiments was also assessed. The study protocol was published on the Open Science Framework prior to testing, where more details about the methods are described (http://doi.org/10.17605/OSF.IO/JBY5X).

### 2.2 Participants

We recruited alcohol consumers using the crowdsourcing platform Prolific Academic (https://www.prolific.ac/). Participants read an information statement before giving their consent to participate. Participants were required to be at least 18 years of age, live in the UK and report drinking alcohol. Prolific Academic was the ideal platform for conducting this study based on the large (n ≈ 8000) number of individuals who regularly use the site who were eligible to participate based on our inclusion criteria. The study was approved by the Faculty of Science Research Ethics Committee at the University of Bristol (reference: 23051753685).

### 2.3 Measures and materials

#### 2.3.1 Demographic questions

Participants were first asked ‘Do you drink alcohol?’. Those who answered “No” were taken to the end of the experiment and not reimbursed. Demographic information included age, gender, and the location of residence within the UK and highest academic qualification. Participants were asked whether they were university students and, if so, what course they study. Problematic alcohol use was assessed using the Alcohol Use Disorders Identification Test (AUDIT) (Bohn et al., 1995a).

#### 2.3.2 Support for alcohol labeling

Participants were asked to what extent they agree with the following statements: 1) ‘Alcoholic beverages should include more information about alcohol strength (i.e., unit information)’, 2) ‘Alcoholic beverages should have information about the health impact of drinking (i.e., health warning labels)’, and 3) ‘Alcoholic beverages should include more nutritional information (i.e., calorie information)’. These questions were answered using a 100-point visual analog scale with the anchors ‘STRONGLY DISAGREE’ and ‘STRONGLY AGREE’.

#### 2.3.3 Unit information task and stimuli

##### 2.3.3.1 Unit labels

Participants were randomly assigned to one of the four-unit label conditions: 1) Basic ABV (Alcohol by Volume refers to the strength of the drink), 2) Responsibility Deal, 3) Food Label Equivalent or 4) Pie Chart. The ‘Basic ABV’ label displayed alcohol strength information, which is legally required on all alcohol labels in the UK. This information was also included in the other conditions. The ‘Responsibility Deal’ label showed the total units per bottle, which is used on many existing labels that adhere to the Responsibility Deal requirements. The novel ‘Food Label Equivalent’ label provided the number of units per serving as a percentage of the low-risk amount, which follows the example of voluntary food labeling schemes in the UK. The novel ‘Pie Chart’ label was designed by the research team and displayed the number of units per serving as a visual proportion of the low-risk amount (14 units per week). The unit label was presented four times to each participant with information corresponding to one of four different alcoholic drinks on each presentation (see Table 1 for details).

**Table 1:** Unit presentation conditions.

	Alcohol bottle stimuli	Unit Label Condition			
		Basic ABV	Responsibility Deal	Food Label Equivalent	Pie chart
Hardy’s – white wine 8.6 units / bottle 2.0 units / 175 serving 7 servings per week					
**Stella Artois – beer** 1.4 units / bottle 1.4 units / 284 ml serving 10 servings per week					
Strongbow – cider 10 units / bottle 2.8 units / 568 ml (pint) serving 5 servings per week					
Smirnoff – vodka 28 units / bottle 1.0 unit / 25 ml serving 14 servings per week					

Note that alcohol bottle stimuli were presented to participants in color.

##### 2.3.3.2 The accuracy of weekly serving limit estimates

To examine if label presentation can improve accuracy when calculating units and weekly drinking guidelines, participants were shown four alcoholic beverages alongside the unit label for each beverage according to their condition (see Table 1). The four beverages reflected the most popular drink types, and brands in the UK and the presentation order of the four beverages were randomized. Participants were asked: ‘How many [serving name (XX ml)] of this [wine/beer/cider/vodka] could you have in a week before reaching the recommended limit of 14 units per week?’ The accuracy of this estimate was the primary outcome measure for the unit information task. Time taken to make the response was also measured (participants were asked to ‘answer as quickly and as accurately as you can’, and told that they must not use a calculator).

##### 2.3.3.3 Drink choice

**Fig. 1:**
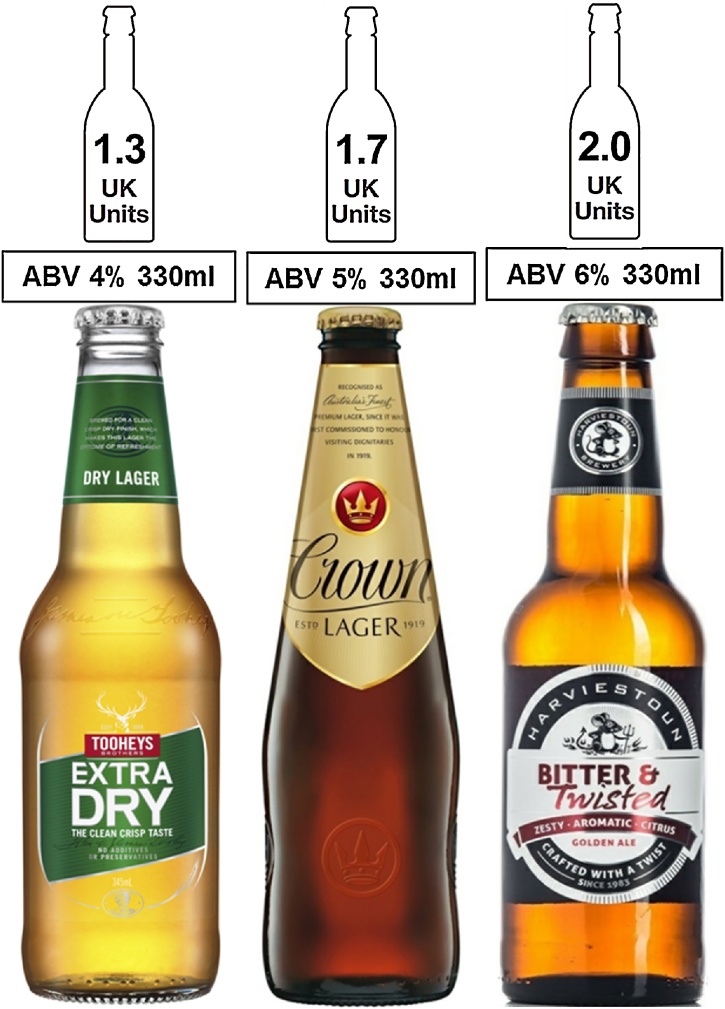
Example beer and Responsibility Deal labels for drink choice task (note that beers were presented to participants in color).

To examine the impact of unit labeling on drink choice, participants were then presented with three bottles of non-UK (i.e., relatively unfamiliar) beer brands, simultaneously on screen, each alongside the unit label as per their randomly assigned condition (see Fig. 1). The three beers were labelled with different alcohol strengths (ABVs): 4%, 5% and 6%, which were randomized between the three beer brands. Participants were asked ‘Which beer would you choose to drink?’. Participants were required to click on one of the beers.

#### 2.3.4 Health warning task and stimuli

Table 2 presents the eight different health warnings used, categorized according to message specificity (general vs.specific), message framing (positive vs.negative) and message content (cancer vs.mental health). Participants were randomly assigned to view one of these warnings towards the bottom of a bottle of unfamiliar beer in black text on a white background.

**Table 2:** Health warning messages.

	General		Specific	
	Negatively framed	Positively framed	Negatively framed	Positively framed
Cancer	Alcohol increases your risk of cancer	Drinking less reduces your risk of cancer	Alcohol increases your risk of bowel cancer	Drinking less reduces your risk of bowel cancer
Mental health	Alcohol increases your risk of mental illness	Drinking less reduces your risk of mental illness	Alcohol increases your risk of depression	Drinking less reduces your risk of depression

##### 2.3.4.1 Motivation to drink less

Participants were asked ‘Does this health warning make you feel motivated to drink less?’ This question was answered on a five-point scale from ‘STRONGLY DISAGREE’ (coded 1) to ‘STRONGLY AGREE’ (coded 5) (adapted from (Wakefield et al., 2017)). This measure has been used to assess responses to anti-alcohol advertisements (Wakefield et al., 2017).

##### 2.3.4.2 Reactance

The Brief Reactance to Health Warnings Scale was administered (Hall et al., 2017). This required participants to rate the extent to which they agree that ‘This warning is trying to manipulate me’, ‘The health effect on this health warning is overblown’ and ‘This warning annoys me’. Statements were scored on a five-point scale from ‘STRONGLY DISAGREE’ (coded 1) to ‘STRONGLY AGREE’ (coded 5).

##### 2.3.4.3 Avoidance

Avoidance was measured with three items, preceded by the text ‘Imagine that all alcohol containers had this warning’ 1) ‘How likely is it that you would try to avoid thinking about the warning?’ 2) ‘How likely is it that you would try to avoid looking at the warning on your drink?’, and 3) ‘How likely is it that you would keep the drink out of sight to avoid looking at the warning?’ Questions were answered on a five-point scale from ‘NOT AT ALL LIKELY’ (coded 1) to ‘EXTREMELY LIKELY’ (coded 5).

##### 2.3.4.4 Believability

Participants were asked ‘How believable is this health warning?’ This question was answered on a five-point scale from ‘NOT AT ALL BELIEVABLE’ (coded 1) to ‘EXTREMELY BELIEVABLE’ (coded 5).

#### 2.3.5 Other measures

To assess self-efficacy, participants were asked ‘For me cutting down on the number of alcohol units that I drink in the next week would be…’ ‘VERY DIFFICULT’ (coded 1) to ‘VERY EASY’ (coded 5). To assess response-efficacy, participants were asked, ‘To what extent do you think that cutting down on your drinking would reduce your risk of alcohol-related disease?’ Questions were answered on a five-point scale from ‘NOT AT ALL LIKELY’ (coded 1) to ‘EXTREMELY LIKELY’ (coded 5). To assess alcohol craving, participants completed the Alcohol Urges Questionnaire (AUQ) (Bohn et al., 1995b).

### 2.4 Procedure

The study was designed and hosted on the Qualtrics online survey platform (http://www.qualtrics.com/). Participants were first shown an information statement and then completed a tick-box consent page. Participants then completed the demographic screening questions and reported their support for alcohol labeling policies. Participants were pseudo-randomized into the different experimental conditions (i.e., one of four-unit information conditions and one of eight health warning conditions) such that an equal number of participants were in each condition. They then completed the unit information and health warning tasks in that order.

Participants completed the self-efficacy and response efficacy questions and the AUQ. This section included one attention check question (‘This is an attention check question, please select the ‘extremely likely’ option’). Finally, participants reported their support for alcohol labeling policies, completed the AUDIT, and provided their educational attainment and student status. The experiment lasted approximately seven minutes, and at the end, participants were debriefed and reimbursed £1.

### 2.5 Statistical analysis

The sample size was calculated based on the primary outcome measure for the health warning task, motivation to drink less. To detect the main effect in motivation to drink less across participants in health warning conditions of 0.5 (SD = 2; based on a 1–5 scale; d = 0.25), at 95% power and an alpha level of 1%, a sample of 1786 participants was required. We aimed to recruit 1900 participants to account for exclusions based on participants failing attention checks.

Estimates of how many servings of each of the four drinks an individual could have in a week before reaching the limit of 14 units were subtracted from the correct answer for each of the four different beverages. These four scores were then averaged to create an overall accuracy variable. Data were analyzed in IMB SPPS Statistics. The full data analysis plan is presented in the study protocol (http://doi.org/10.17605/OSF.IO/JBY5X).

## 3 Results

The data that form the basis of the results are available from the Bristol Research Data Repository, data.bris, at https://doi.org/10.5523/bris.2l19s5lmdoh7k2jc5n8l2ta95i.

### 3.1 Characteristics of participants

Data were collected from 1908 participants. Of these, 24 participants (13 females) were excluded from all analyses for failing the attention check question. Remaining participants (n = 1884) were 50% female, had a mean age of 35 (*SD* = 12) and 83% lived in England, 10% in Scotland, 5% in Wales and 2% in Ireland. 58% of participants’ highest level of education was from a higher education institution, and 13% were currently students. Of these, 71% were studying for undergraduate degrees. The mean AUDIT score was 7 (SD = 5).

### 3.2 Unit information task

#### 3.2.1 Primary outcome measure - accuracy of estimating weekly serving limits of alcohol

**Fig. 2:**
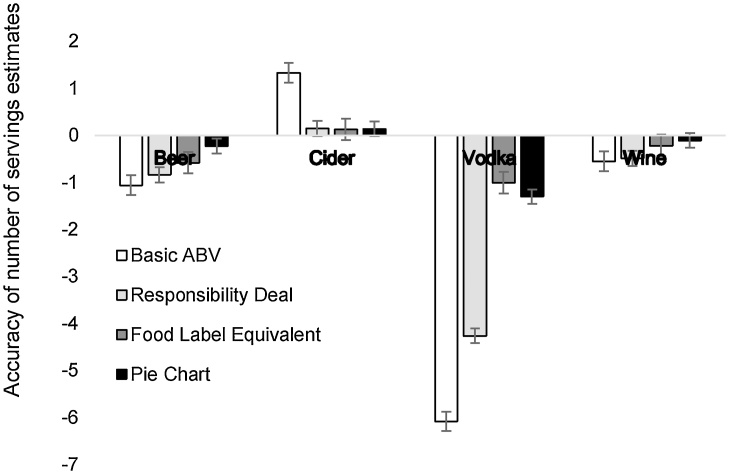
Accuracy of number of servings estimates for the unit information task. Error bars represent standard error of the mean.

As shown in Table 3, the mean accuracy scores for all labeling conditions were below zero, indicating that on average, participants underestimated the number of drinks they could consume within the weekly low-risk guideline of 14 units. One-way ANOVA with unit condition (Basic ABV, Responsibility Deal, Food label Equivalent or Pie Chart) provided evidence for a main effect of unit label condition (F_(3,1880)_ = 22.16 p < 0.001, η^2^ = 0.03). Bonferroni corrected post-hoc t-tests indicated that those in the Food Label Equivalent and Pie Chart conditions were more accurate than those in the Basic ABV and Responsibility Deal conditions (i.e., Responsibility Deal vs. Food Label Equivalent: t_(1880)_ = 4.61, p < 0.001). There were no differences between the estimates for the Basic ABV and Responsibility Deal conditions and between the Food Label Equivalent and Pie Chart conditions. Fig. 2 displays the accuracy estimates across all conditions; participants were particularly inaccurate in estimating the number of servings for vodka. The Shapiro-Wilk test indicated that the data were not normally distributed (p < 0.001) and inspection of the data indicated that there were a large number of data points clustered around zero. Repeating our analyses using the non-parametric Kruskal-Wallis test did not substantially alter our results.

**Table 3:** Summary of all outcome measures for the unit information task.

	Basic ABV (n = 470)	Responsibility Deal (n = 470)	Food Label Equivalent (n = 475)	Pie Chart (n = 469)
Accuracy	−1.60 (3.71)	−1.36 (2.77)	−0.42 (1.98)	−0.38 (2.93)
Time taken	71.12 (54.12)	115.24 (90.59)	83.44 (72.54)	77.01 (46.03)
Self-efficacy	4.21 (0.96)	4.26 (0.94)	4.15 (1.05)	4.17 (1.04)
Response-efficacy	3.73 (1.15)	3.68 (1.18)	3.56 (1.19)	3.55 (1.21)
Alcohol craving (AUQ)	19.16 (7.61)	19.22 (8.19)	20.07 (8.19)	20.23 (9.03)

Figures represent means (standard deviations).

#### 3.2.2 Secondary outcome measures

A series of one-way ANOVAs were conducted with the unit condition on each of the secondary outcome measures (time taken to complete the number of drinks estimates, self-efficacy to drink less, response-efficacy and alcohol craving (AUQ)). A series of Shapiro-Wilk tests indicated that the data were not normally distributed (ps<0.001) and visual inspection confirmed this. We performed Kruskal-Wallis tests on these data, but these did not meaningfully change the findings. As a result, here we report only the results of the ANOVAs, with the means presented in Table 3.

##### 3.2.2.1 Time taken

There was a main effect of time taken to complete the task (F_(3, 1880)_ = 39.30 p < 0.001, η^2^ = 0.06). Participants in the Basic ABV condition completed the unit task in the shortest time followed by the Pie Chart condition and Food Label Equivalent condition. Participants in the Responsibility Deal condition completed the unit task in the slowest time, and post-hoc Bonferroni corrected t-tests indicated that the only evidence of a difference was between the Responsibility Deal condition and the other three conditions (e.g., Responsibility Deal vs.Food Label Equivalent: t_(1880)_ = 6.93, p < 0.001).

##### 3.2.2.2 Self-efficacy

There was relatively high self-efficacy across participants (M = 4.2; SD = 1.0), and there was no evidence of a difference between those in the four conditions (F_(3, 1880)_ = 1.15 p = 0.33, η^2^ = 0.002).

##### 3.2.2.3 Response-efficacy

Overall there was relatively high response-efficacy across participants (M = 3.63; SD = 1.2). There was weak evidence of a difference between those in the four conditions (F_(3, 1880)_ = 2.73 p = 0.04, η^2^ = 0.004) with the highest response-efficacy among those in the Basic ABV condition.

##### 3.2.2.4 AUQ

There was weak evidence of a difference in alcohol craving between those in the four conditions (F_(3, 1880)_ = 2.14 p = 0.09, η^2^ = 0.003).

##### 3.2.2.5 Drink choice

**Fig. 3:**
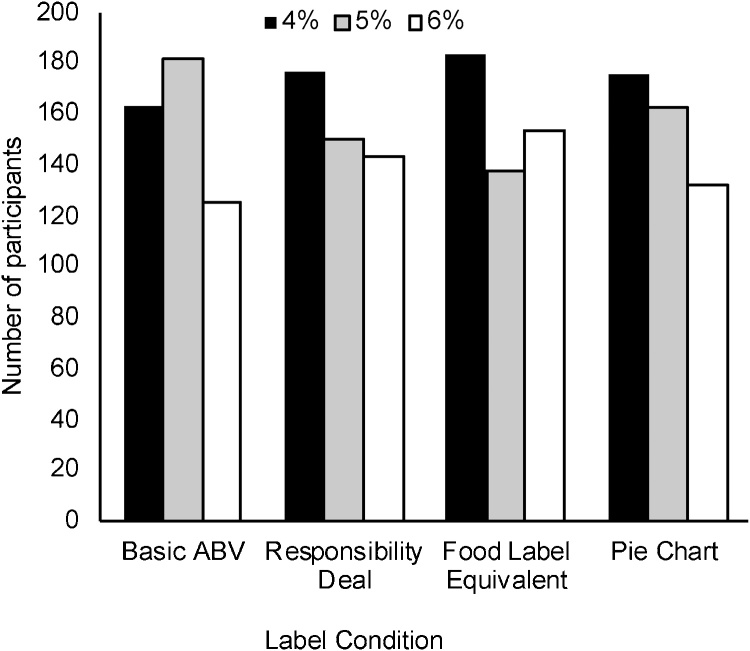
Drink choice for participants in the four-unit label conditions for the unit information task.

An ordinal logistic regression was conducted to examine the impact of the unit condition on alcohol choice (4%, 5% or 6%). There was no evidence that any of the four conditions increased the ordered log odds of choosing a higher strength beverage (log odds =-0.01, 95% Confidence Interval (CI)=−0.08–0.07, p = 0.86; see Fig. 3).

### 3.3 Health warning information

A series of 2 (content) × 2 (specificity) × 2 (framing) factorial ANOVAs were conducted on each of the outcome measures. Visual inspection indicated that the believability, self-efficacy, and response-efficacy data were negatively skewed and the alcohol craving (AUQ) data were positively skewed (Shapiro-Wilk tests p < 0.001). Log-transforming these data and re-running the ANOVAs did not meaningfully change the results. As ANOVA is robust to non-normality, particularly when the sample size is large, we report here only the ANOVA results for non-transformed data. Means are reported in Table 4.

**Table 4:** Summary of outcomes for the health warning information.

	Health warning message			Health warning specificity			Health warning framing		
	Cancer	Mental health	P value	General	Specific	P value	Negative	Positive	P value
Motivation to drink less	2.77 (1.20)	2.63 (1.21)	0.01	2.74 (1.20)	2.67 (1.20)	0.24	2.77 (1.14)	2.63 (1.17)	0.01
Reactance	2.91 (1.08)	2.91 (1.08)	0.91	2.97 (1.08)	2.85 (1.07)	0.01	2.97 (1.08)	2.84 (1.06)	0.02
Avoidance	2.98 (1.17)	2.72 (1.10)	<0.001	2.90 (1.12)	2.81 (1.15)	0.10	2.92 (1.12)	2.79 (1.15)	0.01
Believability	3.48 (1.09)	3.41 (1.08)	0.18	3.34 (1.08)	3.55 (1.07)	<0.001	3.46 (1.10)	3.43 (1.06)	0.53
Self-efficacy	4.16 (1.02)	4.23 (0.98)	0.11	4.17 (1.10)	4.22 (0.98)	0.35	4.19 (1.02)	4.20 (0.98)	0.76
Response efficacy	3.59 (1.19)	3.67 (1.18)	0.17	3.56 (1.19)	3.70 (1.18)	0.01	3.61 (1.20)	3.64 (1.17)	0.59
Alcohol craving (AUQ)	19.60 (8.35)	19.73 (8.21)	0.73	19.69 (8.16)	19.65 (8.40)	0.92	19.53 (7.88)	19.81 (8.67)	0.47

#### 3.3.1 Content - cancer versus mental health warnings

There was evidence that motivation to drink less was higher among those participants randomized to the cancer warning versus mental health warning (F_(1, 1876)_ = 6.45, p = 0.01, η^2^ = 0.003). Avoidance of warnings was also higher among those in this condition (F_(1, 1876)_ = 21.93, p < 0.001, η^2^ = 0.012).

#### 3.3.2 Specificity

General Versus Specific Warnings. There was evidence that those randomized to view the specific warning reported lower levels of reactance (F_(1, 1876)_ = 7.04, p = 0.008, η^2^ = 0.004), found the warning more believable (F_(1, 1876)_ = 16.75, p < 0.001, η^2^ = 0.009) and had higher response-efficacy (F_(1, 1876)_ = 6.94, p = 0.008, η^2^ = 0.004) versus those who viewed a general warning.

#### 3.3.3 Framing

Negative Versus Positive Warnings. The negatively framed warnings received higher scores for motivation to drink less (F_(1, 1876)_ = 7.01, p = 0.008, η^2^ = 0.004), reactance (F_(1, 1876)_ = 5.87, p = 0.015, η^2^ = 0.003) and avoidance (F_(1, 1876)_ = 6.10, p = 0.014, η^2^ = 0.003) than the positively framed warnings.

### 3.4 Support for labeling policies

A 2 (pre-study vs.post-study) × 3 (information type: strength information, health warning, calorie information) ANOVA was conducted to examine change in support for labeling policies. This revealed an interaction (F_(2,3764)_ = 15.07, p < 0.001, η^2^ = 0.008) characterized by an increase in support for strength information (pre: M = 66.8, SD = 26.8, post: M = 69.7, SD = 26.3; t_3766)_ = 8.52, p < 0.001) and calorie information (pre: M = 66.0, SD = 28.1, post: M = 67.2, SD = 28.0; (t_3766)_ = 4.43 p < 0.001) after the experiment, but not health warning information (pre: M = 61.3, SD = 27.9, post: M = 61.7, SD = 28.9; t_3766)_ = 0.91, p = 0.36). A main effect of label type (F_(2,3766)_ = 56.74 p < 0.001, η^2^ = 0.029) indicated that support for strength information was marginally greater than for the calorie information (t_(3766)_ = 1.89 p = 0.06), which in turn was greater than for the health warning information (t_(3766)_ = 7.21, p < 0.001), which had the lowest levels of support.

A series of linear regressions indicated that those with higher levels of support at the beginning of the experiment reported lower levels of reactance (B=-0.13, 95% CI=-0.15 to -0.12, p < 0.001) and avoidance (B=-0.003, 95% CI=-0.005 to -0.001, p = 0.001) and higher levels of warning believability (B = 0.009, 95% CI = 0.007–0.010, p < 0.001), motivation to drink less (B = 0.016, 95% CI = 0.015–0.018, p < 0.001), self-efficacy (B = 0.004, 95% CI = 0.003–0.06, p < 0.001) and response efficacy (B = 0.010, 95% CI = 0.008–0.012, p < 0.001).

## 4 Discussion

Alcohol labeling provides a relatively low-cost, population-level approach to providing consumers with information about the content and harms related to alcohol consumption. The presentation and format of health information can impact on the effectiveness of labels to communicate accurate information and encourage healthier behavior. Overall, participants underestimated the number of drink servings they could have within the low-risk weekly amount of 14 units, which demonstrates the difficulty drinkers have estimating alcohol consumption in units and across multiple drinks. The absence of a difference in accuracy between the UK industry standard Basic ABV and Responsibility Deal conditions and the increased accuracy shown in the novel Food Label Equivalent and Pie Chart conditions demonstrates that current unit labels could be improved. Accuracy of estimating weekly serving limits of alcohol was the least accurate amongst participants in the Responsibility Deal unit condition, as well as the slowest, which suggests that these labels are particularly difficult for consumers to use, despite the intention to provide ‘labels with clear unit content’ (Department of Health, 2011). The information provided on this label related to the total number of units in a bottle, (which corresponded to a single serving for the beer, and multiple servings of the wine, cider, and vodka).

In comparison, information in the Food Label Equivalent and Pie Chart conditions was always per serving. Portman Group guidance recommends that alcohol labels should include units per container as a minimum key element, while units per typical serving are optional (Portman Group, 2017). Our findings indicate that providing unit information per serving may be more informative.

The type of unit label had little impact on participants’ perceived ability to reduce consumption, the impact it would have on their health or their choice of drink. These findings are consistent with our public survey (unpublished results), in which only a small proportion of participants suggested that they would drink less in response to the unit information. However, it is also possible that if consumers were previously underestimating how many drinks constituted 14-units (e.g., thought 14-units is equal to fewer than 14-single measures of a 40% ABV vodka), then they could feel encouraged to *increase* their drinking. Martin-Moreno and colleagues considered this issue in a review of the labeling literature and concluded that the potential for misuse of information was not an adequate reason to withhold it from the public, but presents a strong case for the inclusion of health information (Martin-Moreno et al., 2013). Unit labeling alone may not be sufficient for improving public health. Policymakers should consider the addition of health warnings, which, aside from warnings about drinking when pregnant, are currently missing from voluntary industry labeling (Portman Group, 2017).

Our findings provide suggestions for the type of warnings that could be used. Participants reported higher motivation to drink less after viewing both cancer messages and negatively framed messages. The latter is consistent with previous research that found negatively framed messages could support a reduction in alcohol consumption (Jarvis and Pettigrew, 2013) and research from the tobacco literature which suggests that warnings which elicit negative emotions may be most effective (Cho et al., 2017). We found greater reactance in response to negatively framed messages, and avoidance was higher for both negatively framed and cancer messages. Evidence from the tobacco field suggests that avoidance may be a marker of engagement with warnings (Cho et al., 2016; Thrasher et al., 2016) while reactance may be related to reduced quit intentions (Hall et al., 2016). Future research should examine the extent to which avoidance and reactance of alcohol warnings are related to drinking behavior. The lower levels of reactance and increased believability and response-efficacy after viewing specific, rather than general, messages demonstrate the important role that message format can play in supporting positive responses from drinkers. Our findings suggest that a comprehensive alcohol labeling policy could benefit from a broad range of message content and formatting to maximize reach. Furthermore, increased support for health labeling policies was related to more positive responses to warnings; therefore, opportunities to involve the public in the development of public health policies and ensuring they are clearly communicated may facilitate public support.

Our study has a number of important strengths, including a large sample recruited using Prolific Academic, a crowdsourcing site which has been shown in recent research to produce high-quality data (Palan and Schitter, 2018; Peer et al., 2017). There are also a number of limitations to consider. First, our sample comprises a heterogeneous population of UK drinkers which includes both students and non-students. While this means that our data are applicable to a wide range of drinkers, previous research has found that students may exhibit some unintended behaviors in response to alcohol labels (Jones and Gregory, 2009) and so future research should examine these two populations separately. Second, a limitation inherent to all online research is that we are unable to control the conditions under which individuals participated. This could have compromised the data in a number of ways, including participants misinterpreting their task, participants who completed the study together with another individual, leading to possible spill-over effects and participants not being fully attentive to the questions. We used an attention check question to mitigate against this last problem, although this may not have identified all participants who were failing to engage with the task properly. Third, the unit labels and health warnings were only presented a single time to participants, which contrasts with the repeated exposure to the labels that would occur in real-life. It is possible that some of the immediate responses to the labels we observed here (e.g., reactance) may only be short-lived, and equally, other responses, such as motivation to drink less may only develop after repeated exposure. Finally, the drink choice task assumes that participants’ drink choices are determined by alcohol strength and does not take into account other factors such as taste preference or branding, which also play an important role in choice. Future research should examine to what extent drinkers use strength information to determine drink choice.

## 5 Conclusions

Alcohol labeling can improve understanding of the content of drinks and harms related to consumption. Current unit labels implemented as part of voluntary industry schemes can be improved. Further work is needed to identify the features of optimum labels, but this study suggests that including unit information per serving, as well as in the context of the low-risk drinking guidelines, may be key. Clear and consistent unit labeling could help consumers monitor their drinking and understand the extent to which this differs from the recommended guidelines. This study also supports previous claims that unit information should be presented alongside health messages to discourage riskier drinking behavior. Health messages should aim to maximize population reach and expand on the single pregnancy messages currently used in many industry labels.

## Contributors

O.M.M., K.D., A.K.M.B., A.S.A. and M.M. contributed to the conception and design of the study and contributed to the study protocol. K.D. managed the day-to-day running of the study and testing of participants. O.M.M. performed the data analysis and all authors helped with data interpretation. This manuscript was written by A.K.M.B. and O.M.M. with input from all co-authors. All authors read and approved the final version of the manuscript.

## Role of funding sources

This work was supported by an Alcohol Research UK grant (SG 15/16 222) and an ESRC New Investigator Grant (ES/R003424/1), both awarded to O.M.M. and by the Medical Research Council Integrative Epidemiology Unit at the University of Bristol, which is supported by the Medical Research Council and the University of Bristol (MC_UU_12013/6). The funders had no role in study design, data collection and analysis, decision to publish, or preparation of the manuscript.

## Conflict of interest

No conflict declared.
